# Human Apolipoprotein A-I-Derived Amyloid: Its Association with Atherosclerosis

**DOI:** 10.1371/journal.pone.0022532

**Published:** 2011-07-19

**Authors:** Nahuel A. Ramella, Omar J. Rimoldi, Eduardo D. Prieto, Guillermo R. Schinella, Susana A. Sanchez, María S. Jaureguiberry, María E. Vela, Sergio T. Ferreira, M. Alejandra Tricerri

**Affiliations:** 1 Instituto de Investigaciones Bioquímicas La Plata (INIBIOLP), CCT-CONICET, La Plata, Argentina; 2 Facultad de Ciencias Médicas, Universidad Nacional de La Plata, La Plata, Buenos Aires, Argentina; 3 Laboratory for Fluorescence Dynamics, University of California Irvine, Irvine, California, United States of America; 4 Microscopy Unit, Fundación CNIC-Carlos III, Centro Nacional de Investigaciones Cardiovasculares, Madrid, España; 5 Instituto de Investigaciones Fisicoquímicas Teóricas y Aplicadas (INIFTA), Universidad Nacional de La Plata-CCT-CONICET, La Plata, Argentina; 6 Program in Biochemistry and Cellular Biophysics, Institute of Medical Biochemistry, Federal University of Rio de Janeiro, Rio de Janeiro, Rio de Janeiro, Brazil; University of South Florida, United States of America

## Abstract

Amyloidoses constitute a group of diseases in which soluble proteins aggregate and deposit extracellularly in tissues. Nonhereditary apolipoprotein A-I (apoA-I) amyloid is characterized by deposits of nonvariant protein in atherosclerotic arteries. Despite being common, little is known about the pathogenesis and significance of apoA-I deposition. In this work we investigated by fluorescence and biochemical approaches the impact of a cellular microenvironment associated with chronic inflammation on the folding and pro-amyloidogenic processing of apoA-I. Results showed that mildly acidic pH promotes misfolding, aggregation, and increased binding of apoA-I to extracellular matrix elements, thus favoring protein deposition as amyloid like-complexes. In addition, activated neutrophils and oxidative/proteolytic cleavage of the protein give rise to pro amyloidogenic products. We conclude that, even though apoA-I is not inherently amyloidogenic, it may produce non hereditary amyloidosis as a consequence of the pro-inflammatory microenvironment associated to atherogenesis.

## Introduction

Apolipoprotein A-I is the major protein constituent of human high density lipoproteins (HDLs), which play a key role in reverse cholesterol transport (RCT), shuttling excess of cholesterol (Chol) from the circulation to the liver for catabolism [1]. Even though only ∼5% of the total circulating apoA-I is found in lipid-free or lipid-poor forms [2], it is thought that the highly dynamic catabolism of HDL yields this protein conformation which subsequently acquires lipids, enhancing Chol removal in both physiological [3] and proatherogenic conditions [4]. In addition to its role in lipid homeostasis, apoA-I has been recently shown to exhibit antioxidant and anti-inflammatory properties [5] and to inhibit the aggregation and neurotoxicity of the amyloid-β peptide, the main neurotoxin in Alzheimer's disease [6]. Although the possible association between apolipoproteins and neurodegeneration is unclear, increasing apoA-I concentrations have been reported to correlate with decreasing risk of dementia [7], raising the possibility of a novel role of apoA-I in physiological mechanisms of protection against neurological disorders.

About 60% of the secondary structure of apoA-I is organized in amphipathic α-helices, while the N-terminus is composed of ß-sheets and unstructured residues [8]. Based on thermodynamic and circular dichroism measurements, it has been proposed that lipid-free apoA-I exhibits a molten globule-like state under physiological conditions [9]. This state guarantees the structural plasticity of the protein, which partially unfolds when lipids are released and refolds when lipids are taken up. The structural disorder required to fulfill protein biological functions represents, however, a potential risk of self-aggregating unfolded states. Thus amyloidoses constitute a group of diseases characterized by the conversion of a natively folded protein into a misfolded conformation presenting higher content of ß-sheet secondary structure, which aggregates and deposits causing organ damage and serious morbidity [10] [11]. Protein aggregation is characterized by a remarkable polymorphism, in which oligomers, fibers and amorphous aggregates are found as final products [12].

Changes in apoA-I structure induced by oxidation [13] and proteolysis [14] [15] have been described to impair to different extents its interaction with key proteins involved in RCT and its ability to remove Chol from artery walls [13]. Conceivably, changes in apoA-I structure/stability induced by pathological cellular or extracellular conditions could shift the equilibrium from a folded structure towards a misfolded conformation prone to aggregate in extracellular deposits. Indeed, local deposits of wild type apoA-I have been detected in the pulmonary vasculature of elderly dogs [16], in knee joint menisci, inducing amyloidosis associated with aging [17] and in the aortic intima of elderly individuals [18].

Although the reason why wild-type apoA-I-derived amyloid is associated to atherosclerotic plaques [19] is not known, this fact strongly suggests the importance of the local environment on the mechanism of protein folding. In the present study, we have investigated the effects of specific environmental conditions mimicking a pro-inflammatory milieu on the tendency of apoA-I to misfold and to self-aggregate into insoluble, amyloid-like complexes. Results demonstrate the impact of such environmental agents on the equilibrium between native and aggregation-prone conformational states of apoA-I, suggesting the importance of chronic inflammation to induce non-hereditary apoA-I amyloidosis.

## Materials and Methods

### Materials

Guanidine Hydrochloride (Guanidine Hydrochloride), thioflavin T (ThT), Matrix Metalloproteinase-12 (MMP-12, Catalytic Domain) and Hystopaque were from Sigma Chemical Co. (St Louis, MO). His-purifying resin was from Novagen (Darmstadt, Germany). Heparin (for clinical application) from bovine intestinal mucosa (molecular weight ∼15 kDa) was from Rivero (BA, Argentina). 4,4′-dianilino-1,1′-binaphtyl-5,5′-disulfonic acid, dipotassium salt (bis-ANS) and Alexa Fluor 488 (carboxylic acid, succinimidyl ester, dilithium salt) protein labeling kit were purchased from Molecular Probes (Invitrogen, Carlsbad, CA). All other reagents were of the highest analytical grade available.

### Methods

#### Cloning, expression and purification of wild-type apoA-I

The cDNA for human apoA-I, kindly donated by Dr A. Jonas (University of Illinois at Urbana-Champaign, IL), was inserted into a pET-30 plasmid (Novagen, Madison, WI). Quick Change site directed mutagenesis kit (Stratagene, La Jolla, CA) was used to introduce a modification that created an acid labile Asp–Pro peptide bond between amino acid residues 2 and 3 of apoA-I, allowing specific chemical cleavage of an N-terminal His-Tag fusion peptide [20]. Protein expression and purification were performed as described [20]. Purity of the final protein preparation (checked by denaturing polyacrylamide gel electrophoresis SDS-PAGE) was higher than 95%.

To confirm that the wild-type form behaved as the native protein, structural and functional tests were performed comparing the recombinant protein with apoA-I purified from plasma of healthy donors (not shown). Both, structure and lipid-binding behaviors were indistinguishable between both proteins, and thus we will name the wild-type form as “apoA-I” in this manuscript.

#### Protein denaturation and stability

Chemical denaturation was performed by 2-h incubation of 0.1 mg/mL apoA-I (at pH 7.4, 5.0 or 4.0, obtained using citrate-phosphate McIlvainés buffer) at increasing concentrations of GndHCl at 25°C. Preliminary experiments showed that 2 h were sufficient to reach equilibrium under these conditions. Measurements were performed on an Olis upgraded SLM4800 spectrofluorometer (ISS Inc, Champaign, IL). The free energy of unfolding in the absence of denaturant (ΔG^0^) was obtained from the shift in spectral center of mass of the fluorescence emission of Trp residues, assuming a two-state process as previously described [21], [22].

#### ApoA-I fluorescence quenching by acrylamide

The effect of pH on secondary structure and exposure of aromatic amino acids to the solvent was determined by the analysis of intrinsic fluorescence quenching by acrylamide. ApoA-I emission spectra were acquired in the presence of increasing concentrations of acrylamide (0–0.4 M). After correction for buffer effects, the quenching parameters were calculated using a modified Stern-Volmer equation as [23]: 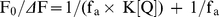
(1)where f_a_ is the fraction of the initial fluorescence which is accessible to the quencher, K is the Stern-Volmer quenching constant of the accessible fraction and [Q] is the concentration of the quencher. F_0_ is the initial fluorescence (contributed by the 4 Trp residues present in apoA-I) and ΔF is the remaining fluorescence after the addition of acrylamide at each concentration. From a linear plot of F_0_/ΔF versus 1/[Q] both K and f_a_ can be obtained.

#### Binding of bis-ANS

ApoA-I (0.1 mg/mL) was incubated at different pH values for 24 h at 37°C. Bis-ANS was then added at a 2∶1 molar ratio (probe:protein), and fluorescence emission was measured on a Beckman DTX 880 Microplate Reader, using excitation and emission filters centered at 395 nm and 490 nm, respectively.

#### Fluorescence correlation spectroscopy (FCS)

FCS measures fluctuations in fluorescence produced when a small number of fluorescent molecules move through an illuminated volume. The fluctuation can be characterized by the autocorrelation function, from which the diffusion coefficient (D_coef_) and the amplitude of the fluctuation G(0) can be readily obtained [24]. For a single species, G(0) is related to the number of particles that move through the illuminated volume by G(0) ∼ γ/Ň, where Ň is the average number of molecules inside the excitation volume and γ is a geometric factor determined by the shape of the point spread function, the mathematical model used and the width parameters [25].

FCS was measured on a two-photon fluorescence microscope at the Laboratory for Fluorescence Dynamics (University of California at Irvine, Irvine, CA), as previously described [26]. Experimental autocorrelation functions were fit using a 3D-Gaussian intensity profile model [26]. Fluorescein was used to calibrate the beam waist of the excitation profile function, considering a D_coef_ of 300 µm^2^/s [27].

#### Detection of amyloid-like aggregates

ApoA-I (0.2 mg/mL) was incubated at different pH values. After 24 h at 37°C, thioflavin T (ThT) was added at a 1∶1 molar ratio and fluorescence intensities were measured on a Microplate Reader, using excitation and emission filters centered at 430 nm and 480 nm, respectively. Plates were then centrifuged at 800 x*g* at 25°C for 30 min, and fluorescence in the supernatant was measured under the same conditions. A set of samples treated exactly under the same conditions was used to quantify total protein remaining in solution after centrifugation using a Qubit Quantitation Platform (Invitrogen, Carlsbad, CA). Sedimented protein was expressed as percentage of the initial amount loaded in each well (20 µg).

#### Binding to heparin

Binding of apoA-I to heparin was followed by light scattering. ApoA-I (0.05 mg/mL) was incubated at different pHs in the absence or presence of heparin (molar ratio 2∶1 heparin:protein for 2 h). Light scattering was monitored at 90° in an Olis upgraded SLM4800 spectrophotofluorometer, with incident light set at 400 nm [28], [29]. In parallel experiments, formation of amyloid-like complexes was followed by incubation of apoA-I (0.2 mg/ml) with heparin for 24 h at 37°C followed by measurement of ThT fluorescence as described above. Integration time was set in each experiment separately. The influence of high salt concentrations on complex formation with heparin was investigated by carrying out incubations in the presence of 500 mM NaCl.

### Pro-inflammatory processing of apoA-I

#### Processing induced by activated neutrophils

Human polymorphonuclear neutrophils (PMNs) were isolated from venous blood of healthy volunteers using a standard method of dextran sedimentation prior to centrifugation in a Ficoll Hypaque gradient and hypotonic lysis of erythrocytes. Purified neutrophils contained >98% viable cells, as determined by trypan blue exclusion. After isolation, PMNs (1×10^5^ cells in 500 µL) were resuspended in Hanks' balanced salt solution (HBSS), pH 7.4, containing 1 mM calcium chloride, 0.5 mM magnesium chloride and 1 mg/mL glucose. ApoA-I (0.2 mg/mL) was added and, after 5 min at 37°C, cells were stimulated with 12-O-tetradecanoylphorbol-13-acetate (TPA) (200 nM), followed by 45 min incubation. Activation was verified by detecting the conversion of nitroblue tetrazolium to formazan due to the neutrophils oxidative burst [30]. Reaction was stopped by spinning the cells at 1,000 x g for 5 min. ApoA-I in the supernatant was then loaded onto a 16% SDS-PAGE gel and developed by western blotting using a polyclonal antibody against apoA-I [31]. An aliquot of apoA-I incubated with PMNs under identical conditions was used to analyze ThT binding.

#### Chlorination of apoA-I in a cell-free system

ApoA-I was dissolved in HBSS, pH 7.4, to obtain a final concentration of 0.2 mg/mL and increasing molar ratios of hypochlorous acid (HClO) were added while vortexing. To ensure that all the HClO had reacted, solutions were left for 1 h at room temperature. The concentration of HClO was determined by measuring the absorbance at 292 nm (ε = 350 M^–1^ cm^–1^) at pH 9.0. Reaction products were analyzed as described above for PMN assays.

#### Proteolysis with metalloproteinase-12(MMP-12)

ApoA-I was incubated with MMP-12 (at a molar ratio 1∶3,000 enzime to apoA-I) at 37°C for 3 h. An aliquot of the reaction mixture was analyzed by SDS-PAGE as described above for PMN assays. In another aliquot, MMP-12 was inhibited by addition of EDTA (final concentration 5 mM), following 24 h incubation at 37°C to determine ThT binding as described above.

#### Chol removal from Chinese Hamster Ovary (CHO) Cells

To check the influence of the oxidative processing on protein function, we incubated apoA-I with HClO for 1 h as described above, and next analyzed the efficiency of the modified protein to solubilize Chol from CHO cells. Cells were grown until confluence, and Chol removal determined as described by Jaureguiberry et al [31]. Efflux was quantified as the percent of the total Chol removed after 12 h incubation with 12 µg/mL of HClO-treated apoA-I in comparison with untreated protein.

#### Other analytical methods

Protein content was quantified by optical density on a Helios β spectrophotometer (Thermo Scientific, Waltham, MA), using an extinction coefficient of 1.13 mL/mg at 280 nm. Transmission electron microscopy was carried out on a JEOL-1200 EX microscope operating at 100 kV. After different incubation periods, samples were centrifuged at 800 x g and the pellet applied onto Formvar-coated grids for 5 min and negatively stained with uranyl acetate (2% solution). For long time incubations of apoA-I, antibiotic-antimycotic mixture (Invitrogen, Carlsbad, CA) was added to the solution. For Atomic Force Microscopy (AFM) analysis, protein under the different treatments was incubated at 0.6 mg/mL for 24 h at 37°C, and spotted stepwise on a freshly cleaved muscovite mica. Following, the residual sample was blotted off with pure water to remove salts, and dried under N_2_. In the case of HClO treatment and control at pH 7.4, a final concentration of 1 mM CaCl_2_ was achieved right before spotting the sample in order to favor protein adhesion to the substrate. All images were obtained in ambient conditions using a Multimode-Nanoscope V (Veeco, Santa Barbara, CA) operating in Tapping Mode with an etched silicon Probe model Arrow-NCR-50 Nano World (cantilever resonance frequency: 258 kHz, Force constant 42 N/m; tip radius 5–10 nm). Typical scan rates were 1 Hz–1.5 Hz.

Unless otherwise stated, experiments are representative of three independent measurements. Results were means ± S.E of at least 3 samples. Statistically significant differences between experimental conditions were evaluated by ANOVA followed by Tukey's test (*p*<0.05).

## Results

### pH effect on the folding and stability of apoA-I

**Figure 1:**
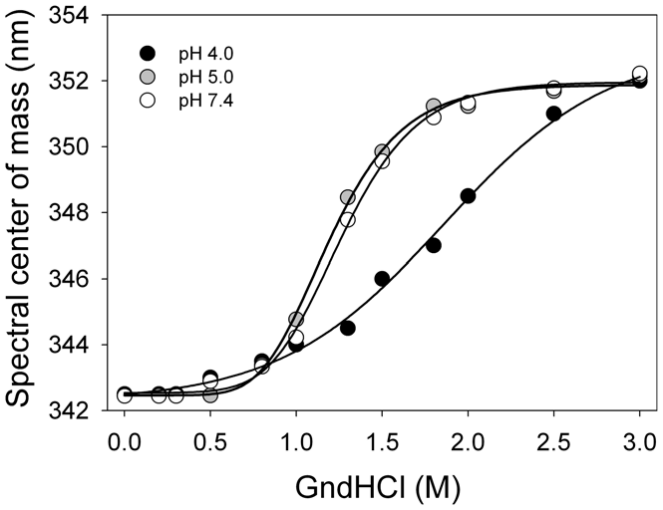
Equilibrium unfolding of apoA-I at different pHs. Spectral centers of mass of the intrinsic fluorescence emission of apoA-I are plotted as a function of [GndHCl]. Final protein concentration was 0.1 mg/mL; excitation was at 295 nm and emission was recorded between 310 and 420 nm. Experiments were performed at pH 4.0 (black), pH 5.0 (gray), or pH 7.4 (white symbols). Lines are fits to the data using a sigmoideal model.

The effect of pH on the structure and stability of apoA-I was investigated in equilibrium unfolding experiments by following the intrinsic fluorescence emission of the protein in the presence of increasing concentrations of GndHCl. The emission spectrum of native apoA-I corresponds to the average signal from four naturally occurring Trp residues (residues number 8, 50, 72 and 108). Shifts in spectral center of mass of the fluorescence emission indicate the average polarity of the environments surrounding the Trp residues in a protein. As previously reported [32], equilibrium at each GndHCl concentration was achieved within a few minutes and the protein was fully unfolded in the presence of 2.0 M GndHCl at pH 7.4 (Figure 1). The equilibrium unfolding transition of apoA-I at pH 7.4 was clearly cooperative and well-defined by a two-state model. The calculated free energy of unfolding was 2.3 kcal/mol (Table 1), which suggests that native apoA-I exhibits a flexible structure likely resembling a molten-globule state [9]. No changes in the denaturation profile were observed at pH 5.0 (Fig 1 gray symbols) compared to 7.4 (empty circles). Instead, at pH 4.0 (Fig. 1 closed symbols) the unfolding transition appeared to be considerably less cooperative. This pattern is no longer defined by a two-state model and thus ΔG^0^ cannot be estimated. The [GndHCl]_1/2_ was displaced to higher concentrations of guanidine and the shifts in Trp exposure to the aqueous medium extended up to 3 M GndHCl. This behavior suggests that at pH 4.0 denaturation of apoA-I proceeds via partially folded intermediate states rather than as a two-state transition.

**Table 1:** Stability and solvent exposure of Trp residues of apoA-I at different pHs.

pH	ΔG^o^ denat^(a)^ (kcal/mol)	K (M^−1^)^(b)^	f_a_^(c)^
7.4	2.3 +−0.3	5.62 +/−0.38	0.66 +/−0.06
5.0	2.2 +−0.4	5.87 +/−0.52	0.73 +/−0.03
4.0	-	7.39 +/−0.39	0.97 +/−0.09

a Free energy change of unfolding calculated from equilibrium unfolding curves is shown in Figure 1 (see “Methods”).

b Stern-Volmer quenching constant (see “Methods”).

c Fraction of accessibility of Trp residues to solvent.

The relative exposure of Trp residues to the aqueous solvent is indicative of protein conformation. To investigate in more detail the solvent accessibility of Trp residues at different pH values, we performed quenching of the intrinsic fluorescence emission by acrylamide. Interaction of Trp residues that are exposed to the aqueous medium with acrylamide results in nonradiative relaxation of the excited state, detected as a decrease in fluorescence intensity [23]. Quenching parameters at the three studied pH values are summarized in Table 1. The quenching constant (K) measured at pH 7.4 (5.62 M^−1^) is in good agreement with a previous report for apoA-I isolated from plasma [33], and with Davidson et al [34] with the difference that their construct included an additional Trp residue in a pro-peptide sequence (position -3). Consistent with results of equilibrium unfolding experiments described above, a similar quenching constant was determined at pH 5.0 (5.87 M^−1^), indicating similar exposure of the Trp residues of apoA-I to the medium. In contrast, at pH 4.0 a higher quenching constant was determined (7.39 M^−1^), suggesting increased exposure of Trp residues to the aqueous medium. By calculating the fraction of fluorescence accessible to the solvent (f_a_) (see “Methods”), and considering that apoA-I contains 4 Trp residues, it was possible to estimate that ∼3 Trp residues (f_a_ ∼0.7) were accessible to the quencher in the native state of the protein (pH 7.4 or 5.0), and that all 4 Trp residues became exposed at pH 4.0 (f_a_ ∼1).

**Figure 2:**
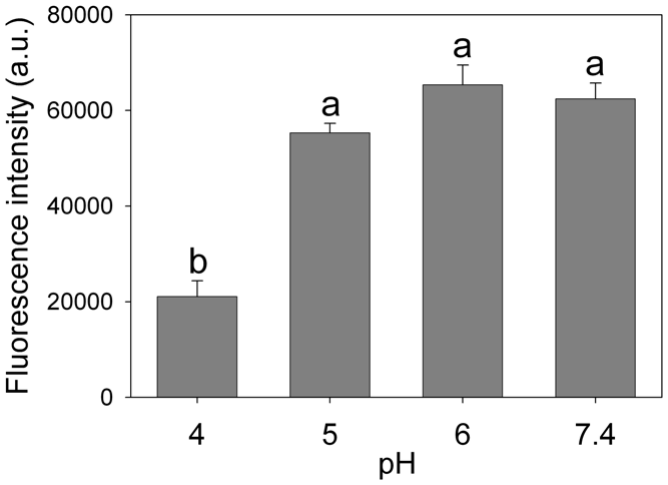
pH dependence on bis-ANS binding to apoA-I. ApoA-I was incubated in optically dark, 96 well microplates, at pH 4.0, 5.0, 6.0 or 7.4 at 37°C for 24 h. Final concentration of apoA-I was 0.1 mg/mL. Bis-ANS was added at a 2∶1 molar ratio to protein, and fluorescence was measured in a Multiplate Reader (excitation at 395 nm, emission at 490 nm). Bars correspond to means ± SE. Bars denoted by different letters differ significantly at p<0.05.

To further examine the influence of pH on apoA-I conformation, we analyzed the binding of the fluorescent probe bis-ANS to the protein. This probe has been widely used to detect surface hydrophobicity of proteins [21], [22], as its fluorescence quantum yield increases markedly upon binding to organized hydrophobic patches at protein surfaces. Figure 2 shows the fluorescence intensity of bis-ANS added to apoA-I previously incubated (for 24 h at 37°C) at different pH values. Binding (detected as an increase of fluorescence) was similar at pH 7.4, 6.0 and 5.0. At pH 4.0, however, the fluorescence intensity was ∼60% lower than that at the other pH values, indicating loss of binding sites for bis-ANS on the apoA-I surface.

Fluorescence Correlation Spectroscopy (FCS) was employed to determine the hydrodynamic properties of apoA-I in a diluted solution at different pH. Measurements were performed at 37°C at the two extremes of pH studied (7.4 and 4.0) using apoA-I labeled with Alexa 488. This probe was chosen because its fluorescence emission is not dependent on pH. The theoretical diffusion coefficient (D_coef_) of a molecule can be calculated using the Stokes-Einstein equation [25]; for apoA-I, with a molecular weight of 28 kDa, this value can be estimated at ∼100 µm^2^/s. From FCS data, experimentally measured D_coef_ values were 119±7 and 98±9 µm^2^/s at pH 7.4 and 4.0, respectively (different with p<0.05). Identical protein concentrations (0.01 mg/mL) were used and the same average number of molecules in the illumination volume was recovered from the G(0) value at both pH values (data not shown). The decrease (by ∼18%) in D_coef_ at pH 4.0 compared to the value obtained at pH 7.4 could reflect either the existence of dimers in solution (a theoretical 20% decrease in D_coef_ would be expected in this case) or the slower diffusion rate of a monomeric protein in an unfolded configuration. Although it is difficult to discriminate between these two possibilities using only D_coef_ as a parameter, the fact that the same G(0) value (number of particles in the sampled volume) was obtained at both pHs is consistent with the interpretation that apoA-I remained monomeric but in a more disorganized (less compact) conformation at pH 4.0. This conclusion is also consistent with the above described results on equilibrium unfolding, fluorescence quenching and bis-ANS binding at different pHs values. Interestingly, incubating the samples for longer periods (∼12 h) resulted in the appearance of large aggregates in the sample at pH 4.0, which were not present at pH 7.4. In addition, some fluorescence was detected when focusing the laser at the bottom of the microscope plate, indicating that acidic pH induced partial sedimentation of the protein during prolonged incubation times.

Taken together, these results suggest that, at physiological pH, apoA-I exhibits a flexible conformation that is largely preserved under mildly acidic conditions (pH 5.0). At pH 4.0 and at low protein concentrations, apoA-I remains monomeric, but it shows typical characteristics of partially folded states, including exposure of Trp residues to the solvent and loss of hydrophobic surface patches and folding cooperativity. The fact that FCS results indicated protein aggregation at longer incubation times suggested that the partially unfolded state of apoA-I induced by acidic pH exhibited increased propensity to undergo self-association, driving us to further investigate the nature of aggregates formed.

### Thioflavin-T (ThT) binding and ultrastructural analysis of apoA-I aggregates

**Figure 3:**
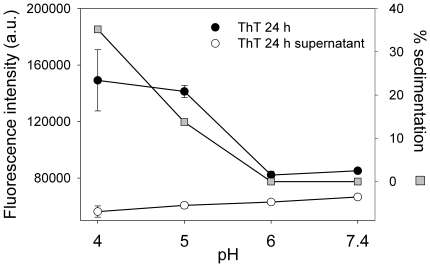
Binding of ThT to apoA-I. ApoA-I was incubated at pH 4.0, 5.0, 6.0 or 7.4 at a final concentration of 0.2 mg/mL at 37°C for 24 h. One µl of ThT (1 mM) was added to each well and ThT fluorescence was measured in a plate reader at 480 nm (excitation at 430 nm) (black circles). Samples were then centrifuged (800 x g for 10 min) and ThT fluorescence remaining in the supernatant (empty circles) was measured. The amount of protein sedimented (gray squares) was calculated as the percentage of the initial protein (20 µg) remaining in solution after low-speed centrifugation. Bars correspond to means ±SE.

Misfolded proteins often give rise to the formation of different types of protein aggregates, including amyloid fibrils and non-fibrillar species such as soluble oligomers, which have been increasingly implicated in a number of important human diseases [12], [35]. To determine whether acidic pH induced amyloid aggregation of misfolded apoA-I, we measured ThT binding to the protein following incubation at different pH values. The fluorescence quantum yield of ThT is very low in aqueous solution, and it increases significantly upon binding of the probe to amyloid aggregates [36]. Although ThT binding usually increases proportionally to the yield of more organized aggregates, significant fluorescence is detected even when proteins are present as oligomeric states [37], [38]. ApoA-I was incubated at different pHs for 24 h and ThT fluorescence was measured before and after low speed centrifugation of the samples (Fig. 3). As expected, ThT fluorescence was very low (similar to the fluorescence of the same amount of ThT in the absence of protein, not shown), at both pH 7.4 and 6.0, conditions in which the protein remained native and soluble after centrifugation. ThT fluorescence increased significantly at pH 5.0 and ∼15% of the protein in the samples sedimented upon centrifugation, revealing the presence of insoluble aggregates. At pH 4.0, ThT binding was also high and even more protein (30–40%) sedimented after centrifugation, indicating that acidification of the medium promotes aggregation of apoA-I to form high molecular weight ThT-binding aggregates. ThT fluorescence in the supernatant was negligible after centrifugation at all pHs tested.

**Figure 4:**
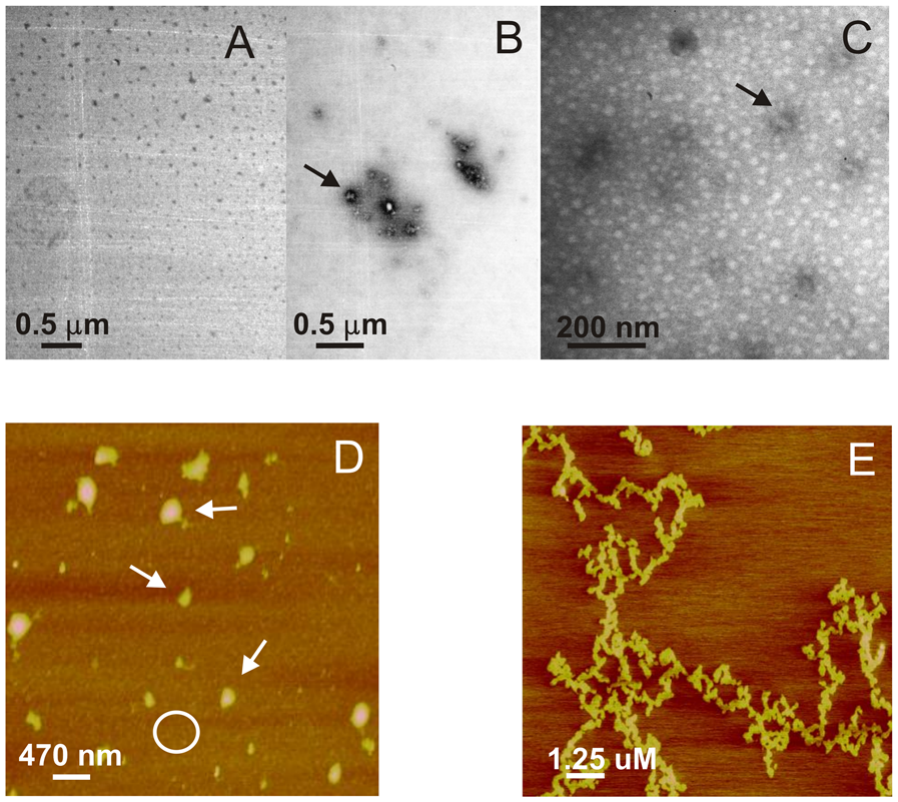
Morphology of pH-induced apoA-I aggregates. Panels illustrate representative images of apoA-I samples studied by transmission Electron Microscopy (A–C) and Atomic Force Microscopy (D,E) A) and B) Protein was incubated at 0.4 mg/mL and 37°C for 24 h at pH 7.4 or pH 5.0, respectively. C) ApoA-I after 48 days incubation at 37°C and pH 5.0. Black arrows in the figures indicate some of the oligomeric structures. D) ApoA-I (0.6 mg/mL) incubated at pH 5.0 for 24 h at 37°C, and loaded onto mica. Small size oligomers covering the surface of the mica (represented by the white circle), plus some larger complexes (white arrows) predominated, and some long, thin, unstructured protofibers could also be observed (panel E). Scale bars are shown in each panel.

We next characterized the morphology of apoA-I aggregates by transmission electron microscopy, after incubating the protein at 0.4 mg/mL and 37°C for 24 h at pH 5.0 and 7.4. As expected, a homogeneous pattern was observed at pH 7.4 after different incubation times, indicating the absence of aggregates (Fig. 4A). Instead, the most conspicuous structures observed at pH 5.0 were small oligomers ranging from 10 to 50 nm in diameter (Fig 4B), similar in size and structure to oligomers reported for other amyloid peptides [39] [40] [41] [12] [42]. Interestingly, these aggregated species were present at different incubation times and organized fibers of typical amyloid morphology were not detected even after 48 days incubation either at pH 5.0 (Fig 4C) or at pH 4.0 (not shown). In order to expand the morphology characterization, we performed AFM analysis of the sample in higher concentrations and overloading the mica with successive applications of the sample. In this condition, the predominant pattern is observed as a background composed of closely packed material of protein oligomers, of an average height ranging between 5 and 10 nm (Fig 4D). In addition, some long, unstructured protofibers appeared (Fig 4E). Control of protein loaded in the same condition at pH 7.4, showed small amount of oligomers scattered on a bare mica (not shown).

Altogether, results indicate that acidic pH alters the delicate equilibrium between the native and self-associating protein structure, inducing the formation of insoluble aggregates. Interestingly, an aggregation-prone conformation of apoA-I is detected in conditions where only mild structural changes are observed (pH 5.0).

### Aggregation induced by heparin binding

**Figure 5:**
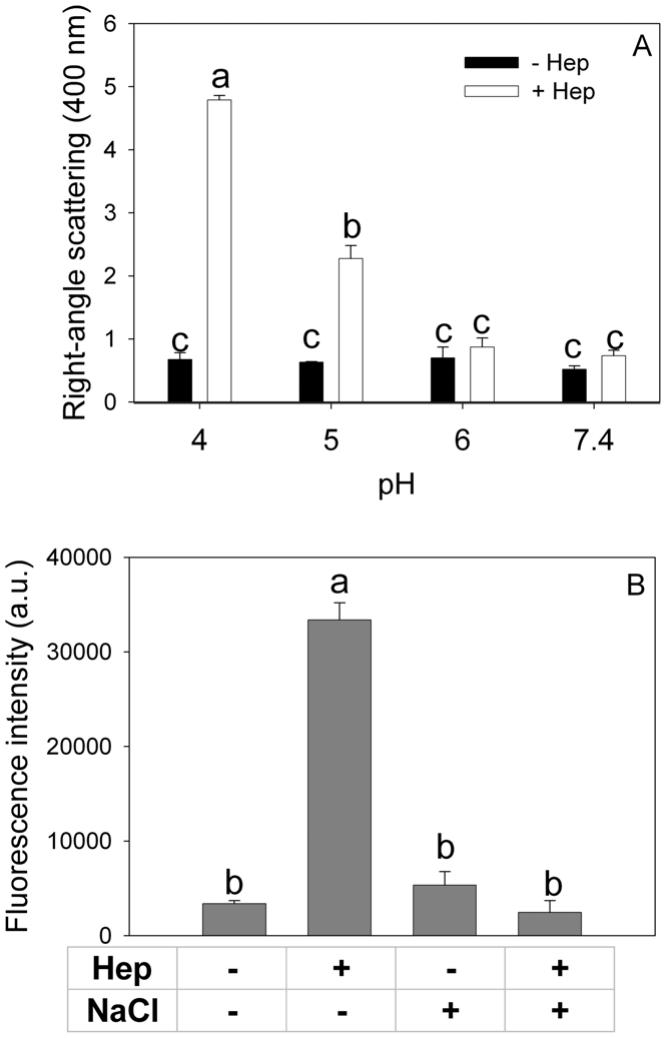
Binding to heparin as a function of pH and salt concentration. (A) Light scattering signal (at 400 nm) of 0.05 mg/mL apoA-I incubated for 2 h at different pH values in the absence (black bars) or in the presence (white bars) of heparin (2∶1 molar ratio heparin:protein). (B) ThT binding to heparin-apoA-I complex at pH 5.0 in the presence or absence of 500 mM ClNa. ThT fluorescence was measured as described in Figure 3. Bars correspond to means ±SE. Bars denoted by different letters differ significantly at p<0.05.

There is abundant evidence that glycosaminoglycans (GAGs) stimulate the formation of amyloid aggregates from different proteins [43], [44]. Binding of heparin and other GAGs to proteins has been shown to depend on protein conformation and on pH [28]. Although it was previously described that apoA-I does not interact with heparin at neutral pH [45], we tested the possibility that binding could be modulated by changes in extracellular pH. Interaction of apoA-I with heparin was analyzed by right-angle light scattering at low protein concentrations. Light scattering is proportional to the size of the molecules in solution, and thus it has been a widely used tool to estimate the formation of high molecular weight complexes in dilute solution [28]. ApoA-I (0.05 mg/mL) was incubated at different pH values with or without heparin (molar ratio 2∶1 heparin:protein) for 2 h, and scattered intensities at 400 nm are shown in Figure 5A. In the absence of heparin, light scattering was low at all pHs investigated, indicating that incubation under those conditions had no significant effect on the aggregation of apoA-I. In contrast, a different behavior was observed in the presence of heparin. At both pH 7.4 and 6.0, light scattering was also low, in agreement with the reported absence of binding sites for heparin on apoA-I at neutral pH. Acidification of the medium at pH 5.0 and, especially, at pH 4.0 caused marked increases in light scattering, indicating that heparin binds to and promotes aggregation of apoA-I at acidic pH. Interestingly, incubation of apoA-I in the presence of heparin at pH 5.0 for 24 h resulted in a marked increase of ThT fluorescence (Fig. 5B), showing an amyloid-like structure of formed aggregates. Addition of 0.5 M NaCl completely blocked this effect, suggesting that interaction between apoA-I and heparin at acidic pH is mediated by salt bridges.

### Influence of oxidative and proteolytic modification of apoA-I on protein folding and function

**Figure 6:**
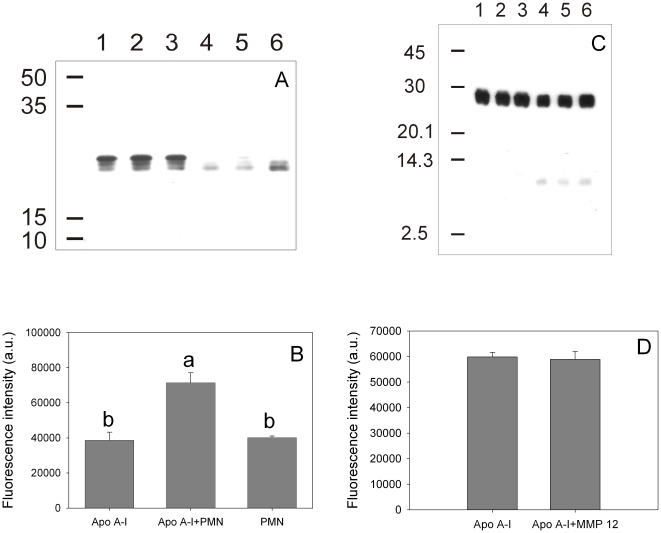
Effect of TPA-activated neutrophils and MMP-12 on apoA-I processing and amyloidogenicity. ApoA-I (0.2 mg/mL) was incubated in the presence of TPA-stimulated neutrophils at pH 7.4 at 37°C for 1 h. (A) Western blotting of aliquots of the supernatant developed with rabbit polyclonal anti-apoA-I. Lanes 1 to 3 show samples incubated in the absence of neutrophils, while lanes 4 to 6 represent the same amount of protein applied to each lane after neutrophil treatment. (B) ThT binding to apoA-I previously incubated (24 h at 37°C) in the absence (labeled as ApoA-I) or in the presence of TPA-stimulated neutrophils (ApoA-I + PMN). As an additional control, ThT fluorescence associated to neutrophils incubated under the same conditions without apoA-I is also shown (bar labeled PMN). In a different experiment apoA-I (0.2 mg/mL) was incubated in the presence of MMP-12. (C) Western blotting was developed as in (A). Numbers on the left side of the figures (A) and (C) correspond to Molecular Weight Markers (GE Healthcare, Uppsala, Sweden). (D) After 3 h at 37°C, MMP-12 was inhibited and apoA-I incubated as in (B) to determine ThT binding. Bars correspond to means ±SE. Bars denoted by different letters differ significantly at p<0.05.

The effect of a pro-inflammatory environment on the structure and function of apoA-I was mimicked by incubating the protein with TPA-stimulated neutrophils, as the activation of these cells is known to lead to complex pathways including oxidative and proteolytic reactions. First, we set up to establish the consequence of such events on protein structure and misfolding. Incubation of apoA-I with activated PMN resulted in partial protein degradation, determined by a decrease of intensity in the 28 kDa band corresponding to the intact protein and appearance of a fragment of ∼22 kDa in SDS-PAGE (Fig. 6A). In some experiments, degradation was considerably more drastic, with complete disappearance of the intact apoA-I band and appearance of high-molecular weight cross-linked products (not shown). Interestingly, partially degraded apoA-I bound significantly more ThT than the intact protein (Fig. 6B), suggesting that PMN-mediated processing of apoA-I gives rise to a pro-amyloidogenic conformation.

Proteolytic degradation has been previously observed upon incubation of apoA-I with macrophages, yielding protein fragments with sizes 26, 22, 14 and 9 kDa, which corresponded to both, the N and C terminus of the protein [14]. As metalloproteinases are known to be highly activated in leukocytes, we checked the effect that metalloproteinase 12 (MMP-12, present in atherosclerotic lesions [46]), could exert on apoA-I pro amiloidogenic processing. Figure 6C shows, as expected, that apoA-I is in some extent substrate of this enzyme, detected as a slight decrease in the intensity of the band associated to original molecular size, together with the appearance of fragments of lower molecular weight; nevertheless, the product does not seem to be amiloidogenic, as the binding to ThT does not change significantly (Fig 6D).

**Figure 7:**
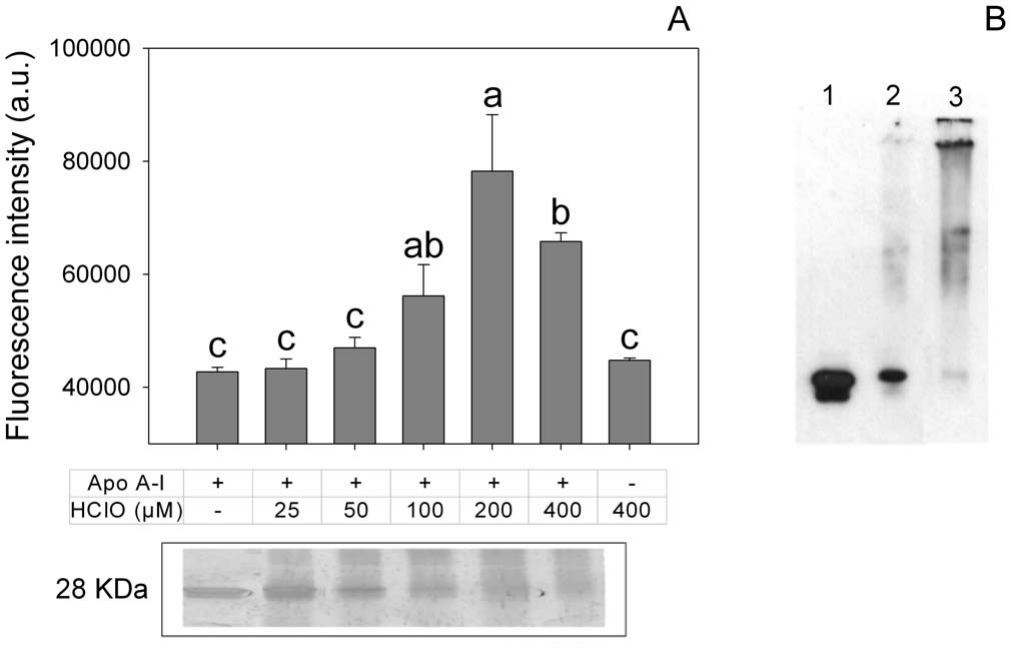
Effect of HClO on apoA-I processing and amyloidogenicity. ApoA-I (0.2 mg/mL) was incubated at increasing concentrations of HClO (as indicated) for 60 min at room temperature. (A) An aliquot (20 µl) of each sample was resolved on SDS-PAGE and silver stained. Control, non-modifed apoA-I is shown in the first lane (lower panel). Another aliquot was further incubated at 37°C for 24 h and ThT binding was measured (upper panel). Bars correspond to means ± SE. Bars denoted by different letters differ significantly at p<0.05. (B) Western blotting (against apoA-I as indicated in Fig. 5) of HClO-treated apoA-I samples. Lanes 1 to 3 correspond to apoA-I incubated with 0, 25 and 200 µM HClO, respectively.

**Figure 8:**
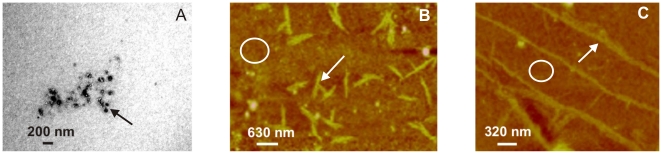
Morphology of HClO-induced apoA-I aggregates. Panels illustrate representative images of apoA-I samples studied by transmission Electron Microscopy (A) and Atomic Force Microscopy (B,C) A) Protein was incubated at 0.2 mg/mL for 24 h and 37°C at 100 µM HClO. Black arrow shows representative aggregates. B and C) ApoA-I (0.6 mg/mL) for 24 h and 37°C at 100 µM HClO, and loaded stepwise onto the mica. Small size oligomers covering the surface of the mica (represented by the white circle), plus some short (B) and long (C) thin protofibers (white arrows) could be observed. Scale bars are shown in each panel.

In addition to proteolysis, different oxidative species are involved in the respiratory burst of activated neutrophils [47]. One of the characteristic responses is the formation of the powerful oxidant and microbicidal agent, HClO, in a reaction catalyzed by myeloperoxidase [48]. To test this effect *in vitro,* apoA-I was incubated at increasing concentrations of HClO and protein integrity was analyzed by SDS-PAGE. Incubation of apoA-I with HClO has been previously tested, and fragmentation of the protein appeared to occur in a random process, as distinct low molecular mass complexes were not detected in that case [49]. Also, in agreement with that report, apoA-I degradation occurred as a function of increasing concentrations of HClO (Fig. 7A, lower panel). To analyze the pro-amyloidogenic processing, we tested ThT fluorescence associated to these products. Interestingly, ThT binding did not linearly correlate with HClO-induced degradation of apoA-I. Instead, ThT fluorescence was maximal at an intermediate HClO:protein molar ratio, but it decreased at higher oxidant concentrations (Fig. 7A, upper panel). Again we characterized these products by microscopy techniques. Samples incubated at 100 µM HClO at low concentrations were observed by electron microscopy as amorphous aggregates (Fig 8A). When incubated at higher concentrations (0.6 mg/mL) and overloaded on the mica, AFM images showed that, in addition to the predominant aggregates, long and short, thin protofibers could be detected in small yield (Fig 8B and C) suggesting that, under more drastic conditions the oligomers could give rise to more organized structures.

These evidences suggest that partial oxidative modification of apoA-I gives rise to amyloidogenic products, whereas further modification by HClO likely results in a more severely unfolded protein conformation that is no longer amyloidogenic. Formation of cross-linked products was also sometimes observed at higher HClO concentrations (Fig. 7B).

**Figure 9:**
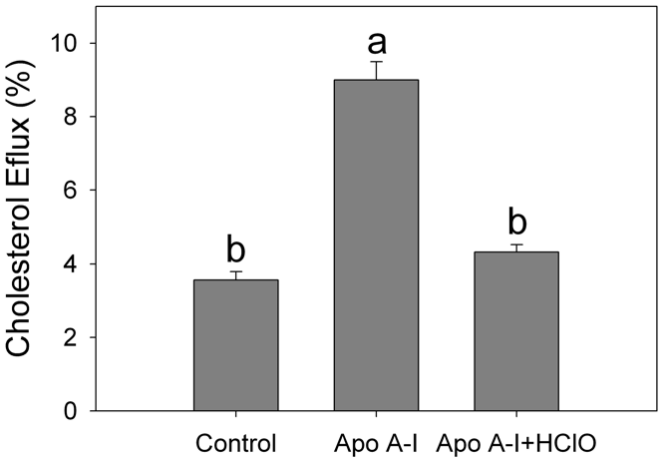
Cholesterol efflux mediated by HClO-processed apoA-I: Chinesse Hamster Ovary Cells were labeled with ^3^H Chol and incubated with apoA-I as indicated in Methods. Results are shown as the % of radioactivity removed by apoA-I, either being treated (ApoA-I + HClO) or not (ApoA-I) with ∼50 µM HClO. Column labeled as Control refers to cells incubated under the same conditions but in the absence of the protein. Bars correspond to means ±SE.

Next, we attempted to characterize the influence of partial oxidation events on the ability of apoA-I to remove Chol from CHO cells. Results showed a significant decrease in protein ability to solubilize Chol (Fig 9), indicating that partial structural modification impairs its ability to participate in cellular Chol homeostasis.

## Discussion

Mutant forms of apoA-I have been involved in late-onset familial amyloidosis [50]. Such mutations are rare and usually associated with systemic deposition of amyloid in tissues, the major clinical features being related to renal, hepatic, and cardiac dysfunction. The possibility that wild-type apoA-I could also be amyloidogenic has been previously raised, as it was localized in senile plaques or associated to atherosclorosis lesions [18], [19]. However, very little is known about the events that might trigger amyloid aggregation of wild-type or mutant forms of apoA-I.

Following its synthesis, apoA-I circulates in plasma and lymph associated to human HDLs and is normally eliminated by filtration in kidney after 4–6 days. Nevertheless, the fact that diffuse deposits of wild type apoA-I are often found associated to age-related and atherosclerotic lesions indicates that, under specific conditions, the protein could lose its structure and aggregate. Thus, both reversible agents (local pH, molecular crowding, interaction with ligands or other biomolecules, etc) and permanent chemical modifications should be taken into account to investigate those factors responsible for aggregation of apoA-I and their possible relationship with amyloidosis.

Atherosclerosis represents a pathological process that underlies the formation of plaques in the intima and media of the arterial wall, resulting from the progressive accumulation of Chol, other oxidized lipids and inflammatory cells. This landscape has been shown to impair HDL and apoA-I function [51]. As activation of inflammatory cells results in a decrease of pH [52], proteolysis and oxidation of specific and unspecific substrates, we have analyzed here the influence of some of these events as possible mediators of apoA-I misfolding.

### Effect of local pH on protein structure and solubility

A critical condition to be considered is the extracelullar pH in the interstitial compartment. Normal oxidative catabolism yields protons which are mostly neutralized by different physiological buffers. However, a local decrease in extracellular pH can occur not only under inflammation [52] but also in chronic hypoxic conditions, which, in addition, induce lactic acidosis.

To investigate the influence of acidification of the medium on apoA-I folding, we exposed the protein to buffers at different pHs. Incubation at pH 6.0 had no effect on the stability and solubility of apoA-I compared to pH 7.4, and no significant binding to ThT was detected, indicating preservation of the native fold. This is not surprising as the iso electric point of apoA-I is 5.27 and thus, the net charge of the protein was largely preserved at pH 6.0. In contrast, at pH 4.0 the structure and stability of apoA-I were significantly modified. Equilibrium unfolding by GndHCl revealed loss of cooperativity in the unfolding transition. Changes in protein conformation were further evidenced by fluorescence quenching, FCS, bis-ANS binding and ThT fluorescence measurements.

Although relevant, results at pH 4.0 are far from *in vivo* landscapes, and thus especial attention should be paid to incubations performed at higher pH. Interestingly, the detection of some increase in ThT fluorescence, along with a decrease in protein solubility and the presence of small aggregates in these samples indicate that the amyloidogenicity of the protein becomes evident at pH 5.0, in spite of the fact that apoA-I retained a mostly native structure at this pH. Similar behavior has been detected for other amyloidogenic proteins, such as transthyretin [53]. From over 70 pro-amyloidogenic natural mutations described for transthyretin, several of them did not show drastic structural changes, except for weaker bonding at protein contacts which increased protein insolubility.

It is also conceivable that at low pH new interactions could be created between apoA-I and other biomolecules in the cellular matrix. As glycosaminoglycans have been shown to stimulate formation of fibrils from different amyloidogenic proteins [**43**], we analyzed the interaction of apoA-I with heparin at different pHs. Results showed that a mild decrease in pH not only generated binding sites for heparin in apoA-I, but it gave rise to formation of complexes with amyloid characteristics (i.e., ThT binding), stabilized by electrostatic interactions.

**Figure 10:**
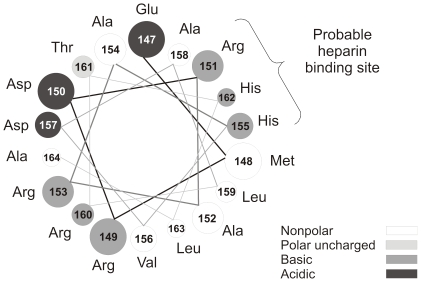
Helical wheel model of putative A α-helix comprised between amino acid residues 147 and 163 of apoA-I. Helix is seen down the long axis, and representing an amino acid arrangement considering an ideal α***-***helix (l00° rotation per residue). Gray scale in the figure corresponds to the nature of different amino acids.

Heparin binding sites in proteins are usually characterized by a cluster of basic residues capable of interacting with the negatively charged heparin polymer. Many different motifs have been postulated as heparin binding domains in proteins [**54**]. Hileman et al. [**55**] have proposed a consensus sequence in which turns from the protein folding bring basic interacting amino acid residues into proximity. The pK_a_ of free histidine is about 6, but it is well-known that ionizable side chains can exhibit changes in their apparent pK_a_, depending on the physicochemical properties of the surrounding protein environment. Thus, it is possible that some histidine residues in apoA-I could become protonated at pH close to 5. ApoA-I has 5 histidine residues (positions 135, 155, 162, 193, 199). Two of them (residues 155 and 162) are located in a putative amphipatic class A α-helix, separated by a periodicity that allows a turn of the helix to bring them in close proximity, and near an arginine residue that is seven amino acids apart in the same helix. By considering the helix-wheel model (Fig. 10), this spatial sequence of 3 amino acids is located in the polar face adjacent to the non polar face in the helix 6. Thus, protonation of the His residues would increase the concentration of positive charges on the polar side of the helix, favoring interaction with negatively charged membranes (like in apoptotic cells) or components of the extracellular matrix.

### Effect of chemical modifications on apoA-I

Our findings strongly suggest that chemical modification of apoA-I induced by conditions mimicking a pro-inflammatory environment results in the processing of apoA-I into partially degraded, amyloidogenic products. As metalloproteinases are highly activated in atherosclerotic plaques [2], we checked whether the incubation of apoA-I with MMP-12 could result in the formation of a peptide with amyloidogenic properties. As expected apoA-I degradation was observed, but this event did not seem to be responsible as the product is not amyloidogenic. Nevertheless, it could be possible that other peptide could be freed under proteolytic processing, as apoA-I hereditary amyloidosis is usually characterized by the presence of N-terminal fragments of the protein in the lesions. By purifying one of these fragments, Andreola et al [56] demonstrated that the acidification of the medium induced the shift from unordered to β sheet conformation and the formation of fibrils from the isolated peptide.

Instead, oxidation is likely to be critical in the pathological processing of the protein, as myeloperoxidase, an enzyme present in polymorphonuclear cells, and its products, as HClO, have been directly involved at different stages of atherosclerotic lesions [57]. When incubating apoA-I with HClO, we showed that the protein was extensively degraded (Fig 5). Degradation included proteolysis and probably oxidation mediated by HClO, as methionine [13], tyrosine [58] and tryptophan [59] residues have been shown to react with myeloperoxidase-generated HClO. Interestingly, partially degraded apoA-I showed a significant ThT-associated fluorescence, indicating a product more prone to aggregate into an amyloid form than the native protein (Fig 6). This observation is really important, as it shows a clear demonstration of the relationship between atherosclerosis and apoA-I-induced amyloidosis. In addition, the oxidized protein loses its ability to remove Chol. This fact could result in the accumulation of Chol in peripheral cells, which is in fact a signal to induce apoptosis, leading to exposure of phosphatidylserine in the outer leaflet of the plasma membrane. Presence of negatively charged lipids in the plasma membrane can induce further local acidification of the pH [60] in the interstitial fluid, which in turn could elicit the conformational shift of apoA-I into a pathological misfolding.

A lower pH induced by pro-inflammatory conditions could also increase binding of apoA-I to the extracellular matrix, extending the time during which apoA-I and HDLs are exposed to macrophage-mediated oxidative damage.

In conclusion, our results strongly suggest that different events taking place in the inflammatory hallmark of atherosclerosis conduct to a pro-amyloidogenic processing of apoA-I which in turn could impair the vascular disease. Although most of the apoA-I circulates associated to HDL, the protein in the lipid-bound state is more stable and protected from the chemical processing, and thus the lipid-poor or lipid-free conformation is the candidate to induce amyloidogenic products. The fact that macrophages increase the yield of this conformation locally at the artery wall [4] supports this concept. Furthermore, the concentration of lipid-free apoA-I in the aortic intima has been shown to increase during the progression of atherosclerosis [61].

Finally, although not tested here, the amyloidogenic conformations of apoA-I are likely to perpetuate the vascular disease as the protein pathological conformation could be cytotoxic and elicit the inflammatory environment. Further research will be done on this topic.
